# Cost comparison of school-based mass drug administration of albendazole and ivermectin versus albendazole alone for soil-transmitted helminth control in Uganda

**DOI:** 10.1371/journal.pntd.0013913

**Published:** 2026-01-14

**Authors:** Yuling Lin, Tanja Barth-Jaeggi, Eveline Hürlimann, Prudence Beinamaryo, Hilda Kyarisiima, Harsh Vivek Harkare, Leonsio Matagi, Isaac Byarugaba, Peter Steinmann, Jennifer Keiser, Fabrizio Tediosi

**Affiliations:** 1 Swiss Tropical and Public Health Institute, Allschwil, Switzerland; 2 University of Basel, Basel, Switzerland; 3 Vector Borne and Neglected Tropical Diseases Division, Ministry of Health, Kampala, Uganda; 4 College of Humanities and Social Sciences, Makerere University, Kampala, Uganda; The University of Melbourne, AUSTRALIA

## Abstract

The co-administration of albendazole and ivermectin (ALB-IVM) is recommended for the treatment of soil-transmitted helminth (STH) infections, especially where the prevalence of *Trichuris trichiura* is high. Before large-scale implementation can be considered, feasibility, acceptability and required resources and investments for the mass drug administration (MDA) of ALB-IVM compared with albendazole (ALB) alone should be assessed. This study, conducted in two districts in south-western Uganda, aimed to assess and compare the costs of school-based MDA with ALB-IVM (MDA-ALB-IVM) *versus* the routinely used ALB alone (MDA-ALB) on a small scale (targeting around 2,500 children per treatment arm per district). We applied a micro-costing (mixed top-down and bottom-up) approach to assess the financial costs from a health system perspective, as well as the opportunity costs of donated ALB. The total (financial and opportunity) costs of MDA-ALB-IVM were higher than those of MDA-ALB ($10,793 for MDA-ALB-IVM versus $4,458 for MDA-ALB in Kabale district and $14,445 versus $5,765 in Kisoro, respectively). The presence of informed consent and assent required for MDA-ALB-IVM (as ALB-IVM is still considered a new therapy for STH infections in Uganda) increased the number of days and resources including personnel requirements for training and drug distribution. Furthermore, adequate community sensitization and the involvement of community health workers (i.e., village health teams in Uganda) and local community leaders appeared to be essential to achieve high treatment coverage. The scenario analysis showed that, in the absence of the informed consent and assent process, the total incremental costs of MDA-ALB-IVM compared with MDA-ALB could decrease by 31%-36% in the two districts. This study identifies key cost drivers and offers insights for the wider implementation of ALB-IVM co-administration.

## Author summary

The combination of albendazole and ivermectin (ALB-IVM) shows higher efficacy against *Trichuris trichiura* compared with albendazole (ALB) alone. This combination is recommended for the treatment of STH infections, especially where *T. trichiura* infection is common. ALB is provided as a single fixed dose, while IVM is dosed using a dose pole to approximate height ranges to weight-based dosing ranges. The different dosing procedures may impact training needs and drug distribution procedures. Before the larger-scale distribution of the combination treatment is implemented, it should be investigated what additional resources and investments are required for ALB-IVM co-administration compared with ALB alone. In this study, we assessed the costs of ALB-IVM co-administration versus ALB alone on a small scale (20 schools in south-western Uganda). The findings showed higher costs of ALB-IVM co-administration than ALB alone. The incremental costs were mainly due to additional resources and day(s) required for training and drug distribution. The study also found that good communication with communities and involving community health workers and local leaders might be important to achieve high treatment coverage. In conclusion, this study highlights the key cost factors and provides insights to support wider rollout of ALB-IVM co-administration.

## Introduction

Soil-transmitted helminths (STHs) infect humans through ingestion of infective eggs (*Ascaris lumbricoides*, *Trichuris trichiura*) or skin penetration by larvae (*Necator americanus*, *Ancylostoma duodenale, Strongyloides stercoralis*) [1]. Globally, it was estimated that 876 million children required preventive chemotherapy in 2023 for combatting STHs [2]. In Uganda, the prevalence of STH infections has been reduced from over 50% in school-aged children (SAC) between 1998–2005 [3] to 9% in 2016 [4]. However, the prevalence remains high in some districts, and this could be because *T. trichiura* (against which current treatment has limited efficacy) is more prevalent while there is a lack of effective delivery channels in these areas [5,6]. For example, in south-western Uganda, the prevalence of any STH infection was around 49–61% in Kisoro and 16–23% in Rubanda, with *T. trichiura* and *A. lumbricoides* infections being the most common species [5].

Mass drug administration (MDA) with albendazole (ALB) or mebendazole is the main strategy for STH control. However, both drugs show limited efficacy against *T. trichiura* [7]. Drug combinations have been explored and have shown improved efficacy [8–10]. Therefore, the 21^st^ meeting of the World Health Organization’s (WHO) Expert Committee on the Selection and Use of Essential Medicines recommended the co-administration of ALB and ivermectin (ALB-IVM) for treatment of STH infections [11].

It is important to assess what additional resources and investments are required for MDA of ALB-IVM (MDA-ALB-IVM) compared with routine MDA with ALB alone to inform resource allocation and mobilization. While there are cost studies evaluating or comparing community-based and school-based MDA [12–14] and assessing the costs of integrated MDA [13,15–17], there is a lack of research assessing and comparing the costs of mass administration of ALB-IVM versus ALB alone for STH infections. ALB is provided as a fixed single oral dose in form of one 400 mg tablet while IVM is dosed based on body weight (200 µg/kg) or in MDA using a dose-pole that approximates height bands to weight-based dosing ranges [18,19]. These differing dosing procedures may affect drug distribution procedures, training requirements, and delivery costs.

As part of the *Feasibility And Cost-Effectiveness of Improved Treatment against helminthiases in children* (FACE-IT) project, an efficacy trial was conducted in Uganda in 2023. This trial showed superior efficacy of ALB-IVM against *T. trichiura* compared with ALB alone (cure rate: 31.3% versus 12.3%; egg reduction rates: 91.4% versus 52.7%) [20].

Following the efficacy trial, an implementation pilot study was conducted in 2024 to assess the feasibility and acceptability of integrating ALB-IVM co-administration into routine school-based MDA to control STH infections. The pilot was implemented across 20 schools in south-western Uganda and included an economic evaluation to assess the costs associated with delivering MDA-ALB-IVM relative to MDA-ALB.

This manuscript presents a descriptive analysis of the cost data from the implementation pilot, detailing the total and disaggregated costs, identifying the principal cost drivers for each MDA strategy, and estimating the incremental costs associated with the introduction of ALB-IVM co-administration.

## Methods

### Ethics statement

Ethical approvals were obtained from the Ethics Committee of North-western and Central Switzerland (no. AO_2024–00035), Makerere University School of Social Sciences Research Ethics Committee (no. MAKSSREC 05.2024.737), and Uganda National Council for Science and Technology (no. SS2755ES).

The study compared MDA-ALB-IVM with the routine MDA-ALB. The MDA-ALB was implemented as closely as possible to the standard national MDA for STH, for which individual informed consent is not required. For schools assigned to MDA-ALB, written consent was sought at the school level and signed by the head teacher.

For MDA-ALB-IVM, given the experimental nature of the implementation pilot and that ALB-IVM was a new treatment for STH infections in Uganda, individual informed written consent was obtained from parents of children who received the combination, complemented by informed written assent from children aged 8 years and above.

### Study setting and population

In Uganda, routine deworming campaigns to control STH infections are conducted twice per year, usually in April and October. This study was implemented in 20 primary schools located in Kabale and Kisoro districts in south-western Uganda in June and July 2024. In each district, ten schools were randomly pre-selected from schools with an enrollment of about 500 pupils according to district-provided enrollment lists. School children from these schools were excluded from the routine MDA campaign for STH infections in April 2024 and instead received treatment as part of the pilot study. The targeted number of children in each treatment arm and district depended on the actual school enrollment numbers (varying between 227 and 1231). Children aged 5–14 years were eligible for the study in accordance with the target age range of the national school-based deworming campaigns [21].

### Sensitization and training

Prior to the treatment interventions, an advocacy meeting was held between a team from the Ugandan Ministry of Health (MoH) and districts’ local governments. In each district, the MoH staff (central supervisors) provided training to district health workers (engaged from the respective health facilities of the catchment areas) and school teachers on different aspects about the disease, treatment and documentation procedures to be applied. After the training, teachers informed and mobilized children to participate in the MDA campaign.

MDA activities were carried out sequentially – first in Kabale district and followed by Kisoro. To prevent or mitigate a certain reluctance observed in Kabale (primarily related to informed consent and assent in certain schools receiving ALB-IVM), additional sensitization efforts were implemented in Kisoro. These included the following measures: (i) broadcasting of audio announcements about the MDA campaign before and during the drug distribution days to sensitize communities (similar to what would be done for routine MDA in Uganda); (ii) involvement and participation of village health teams (VHTs), the assistant district health officer, and the district education officer in the training; and (iii) involvement of VHTs and local community leaders (i.e., elected chairpersons at village level) in community sensitization and social mobilization for drug distribution.

### Interventions

In each district, the ten pre-selected schools were randomly assigned at a ratio of 1:1 to one of the two treatment arms: MDA-ALB and MDA-ALB-IVM. ALB (400 mg) was obtained from WHO (donated by GlaxoSmithKline) and one tablet was administered per child. IVM (3 mg) was purchased from Laboratorios Liconsa, Spain, and administered at a dose of 200 µg/kg. The dose was estimated with a dose pole (i.e., 90–119 cm: 1 tablet of 3 mg IVM, 120–140 cm: 2 tablets, 141–159 cm: 3 tablets, or > 159 cm: 4 tablets) according to WHO guidelines [18,19,22].

Children aged 5–14 years were eligible to participate in this pilot and were treated with the respective medicines depending on which arm the schools were assigned to. In order not to leave anyone behind, children under 5 years received the routine MDA intervention (ALB alone), while children aged 15 years and above received the treatment assigned to the schools in Kabale and ALB alone in all schools in Kisoro (decisions made by the drug distribution team).

In either treatment arm, SAC who declined treatment or whose headteacher or parents did not provide consent, did not receive any medication (either ALB or ALB-IVM) during this MDA campaign.

Drugs were administered by health workers, with teachers assisting in documentation and organizational aspects (e.g., dose determination using a dose pole for IVM, children/class arrangement, and observing tablet swallowing). Central supervisors oversaw the entire process and/or supported wherever needed during drug distribution. In Kisoro, in addition to teachers, VHTs were engaged in both arms for sensitization and mobilization, while local community leaders further supported mobilization in MDA-ALB-IVM. A detailed description of MDA activities is presented in Table A in S1 Text.

Since ALB-IVM was a new treatment regimen for STH infections and the consent procedure was different for each treatment arm, more personnel were involved and more drug distribution days were planned for MDA-ALB-IVM compared with MDA-ALB (Table B in S1 Text). For MDA-ALB, the drug distribution team was split into five groups and treated SAC in all five schools in one day, whereas for MDA-ALB-IVM, the entire drug distribution team went to one school per day so treatment lasted five days.

### Data collection

We assessed the costs of school-based MDA from a programmatic perspective of the health system. A micro-costing approach [23] was applied to identify and measure resources required and a mixed top-down and bottom-up approach was used to value the costs [24,25]. We used an activity table to identify the required resources. We reviewed the actual expenditure documentation to extract financial cost data and contacted MoH staff with structured questions to collect and/or verify information on unit prices, unit quantities, number of personnel and number of activity days. The time required for treatment (the start and end time points of treatment) in schools was collected on tally sheets. For MDA-ALB-IVM, the treatment time included the time required for assenting, as child assent and treatment were conducted sequentially, making it difficult to separate the assent time from the treatment time (children aged eight years and above received treatment immediately after assenting, and children aged under eight years received treatment when their name and class matched their signed parental consent forms). A costing template was used to assemble the collected data.

Refreshments provided during MDA-ALB-IVM and costs of printing consent and assent forms were excluded from the cost analysis. The exclusion of additional personnel requirements and drug distribution days due to the informed consent and assent process was described and explored in the scenario analysis.

The total number of eligible children aged 5–14 years and the number of children treated were counted by teachers or the drug distribution team and documented on tally sheets.

### Cost valuation and allocation

We assessed the financial costs of MDA-ALB-IVM and MDA-ALB, as well as the opportunity costs of the donated ALB tablets. Financial costs were the expenditures for the implementation of MDA, including expenses for purchasing resources required and per diems and allowances given to personnel involved in the implementation (Tables C-D in S1 Text). The opportunity costs of the donated ALB were calculated with a unit price of 0.0385 United States dollars (USD, $) per tablet ($0.035 per tablet – price inquired from the International Dispensary Association Foundation [26] – plus an additional 10% for shipping costs). IVM was valued at $0.117172 per tablet ($0.10652 per tablet – the price at which IVM was purchased for this study in 2024, which is the same price that WHO would pay for purchasing generic IVM from Laboratorios Liconsa, Spain – plus an additional 10% for shipping costs). The IVM costs were categorized as financial costs, given it was purchased for this study. All other financial costs were in 2024 Ugandan shillings (UGX). These were converted to 2024 USD using the annual exchange rate of 1 UGX = 0.0002662 USD [27].

The opportunity costs of personnel time spent by all personnel on all MDA activities were not valued or included in the analysis as data on salary estimates were not collected. However, treatment time in schools and the number of days required for each MDA activity (Table C in S1 Text) were recorded.

MDA activities included advocacy, training, community sensitization, and drug distribution (treatment). Costs of MDA were categorized into five categories: medicines, personnel, transport, supplies and services, and overheads (program administration and running costs) (Table D in S1 Text). Costs were allocated by districts, MDA activities and treatment arms. Fuel costs in districts were allocated to each treatment arm based on approximate travel distances. Training costs were allocated as one day of training for MDA-ALB and two days for MDA-ALB-IVM. Other shared costs were allocated based on the number of activity days or at a ratio of 0.5:0.5 where applicable (Table E in S1 Text).

### Data analysis

The target population size was calculated as the total number of eligible children (aged 5–14 years) enrolled in the intervention schools. The treatment coverage was calculated as the number of eligible children treated divided by the size of the target population. The total costs presented in this study were the sum of financial costs and opportunity costs (of donated ALB). We did not refer to the total costs as economic costs to reflect that the opportunity costs of personnel time were not included in the analysis.

Cost per child treated was calculated by dividing the total (financial and opportunity) costs by the number of eligible children treated. Incremental costs represented the additional costs of MDA-ALB-IVM compared with MDA-ALB, calculated as the total (financial and opportunity) costs of MDA-ALB-IVM subtracted by those of MDA-ALB.

The average time required for treatment in schools was calculated by dividing the total treatment time by the number of schools. The average time per 100 children treated was calculated by dividing the total treatment time by the number of eligible children treated and multiplying by 100.

### Scenario analysis

The presence of an informed consent and assent process affected the organization of drug distribution, particularly the number of days for drug distribution and the number of personnel required per school. These organizational factors influenced the incremental costs between the two interventions. To estimate the costs in the absence of an informed consent and assent process, we explored one scenario (in consultation with the MoH team) where we assumed that in the absence of informed consent and assent processes, the drug distribution team with reduced personnel treated SAC in one to two schools per day (three days for five schools) in MDA-ALB-IVM, while the organization in MDA-ALB remained unchanged – treating SAC in all five schools within one day (Table F in S1 Text). We thus assumed a reduced number of days for drug distribution and reduced personnel requirements in MDA-ALB-IVM, while treating the same number of children. The cost valuation and allocation for some resource items (e.g., car hiring, fuel costs, allowances, and per diems) required for drug distribution were adjusted accordingly.

We present both pilot-based costs (representing costs observed in the implementation pilot) and scenario-adjusted costs.

### Sensitivity analysis

We conducted sensitivity analyses to assess how variations in drug pricing (by halving and doubling drug prices) and changes in treatment coverage (reductions of 10% and 20%) would influence the estimated cost per child treated. In sensitivity analyses for MDA-ALB-IVM in Kabale, we analyzed the impact of reducing coverage by 10% and 20% in schools with ≥95% coverage and increasing coverage by 10% and 20% in schools with <50% coverage.

## Results

### Total and incremental costs

Table 1 presents the pilot-based costs. We observed substantially higher total (financial and opportunity) costs of MDA-ALB-IVM compared with MDA-ALB: $10,793 versus $4,458 in Kabale, and $14,445 versus $5,765 in Kisoro. Across both arms, costs were consistently higher in Kisoro than in Kabale. In both districts, the largest cost shares were from training and drug distribution. For MDA-ALB, training accounted for the largest share of total costs (48.2-49.6%), whereas for MDA-ALB-IVM, drug distribution (including financial and opportunity costs) was the largest share (43.8-47.9%). A breakdown by activities and cost categories revealed that personnel, along with supplies and services, contributed the most to training costs. For drug distribution, personnel and transport were the primary cost drivers.

**Table 1:** Pilot-based total and incremental costs (US$).

Activity/ cost category	Kabale			Kisoro		
	Total costs (%)		Incremental costs (%)	Total costs (%)		Incremental costs (%)
	MDA-ALB	MDA-ALB-IVM		MDA-ALB	MDA-ALB-IVM	
**Advocacy (financial)**	**425 (9.5)**	**425 (3.9)**	**0 (0.0)**	**425 (7.4)**	**425 (2.9)**	**0 (0.0)**
Personnel	200 (4.5)	200 (1.8)	0 (0.0)	200 (3.5)	200 (1.4)	0 (0.0)
Transport	226 (5.1)	226 (2.1)	0 (0.0)	226 (3.9)	226 (1.6)	0 (0.0)
**Training (financial)**	**2,212 (49.6)**	**4,255 (39.4)**	**2,042 (32.2)**	**2,777 (48.2)**	**5,347 (37.0)**	**2,570 (29.6)**
Personnel	941 (21.1)	1,862 (17.2)	921 (14.5)	1,114 (19.3)	2,208 (15.3)	1,094 (12.6)
Transport	311 (7.0)	546 (5.1)	235 (3.7)	434 (7.5)	768 (5.3)	334 (3.8)
Supplies and services	960 (21.5)	1,847 (17.1)	887 (14.0)	1,230 (21.3)	2,372 (16.4)	1,142 (13.2)
**Sensitization (financial)**	**0 (0.0)**	**0 (0.0)**	**0 (0.0)**	**213 (3.7)**	**1,065 (7.4)**	**852 (9.8)**
Supplies and services	0 (0.0)	0 (0.0)	0 (0.0)	213 (3.7)	1,065 (7.4)	852 (9.8)
**Drug distribution (financial)**	**1,337 (30.0)**	**5,116 (47.4)**	**3,780 (59.7)**	**1,726 (29.9)**	**6,275 (43.4)**	**4,549 (52.4)**
Medicines (IVM)	0 (0.0)	348 (3.2)	348 (5.5)	0 (0.0)	346 (2.4)	346 (4.0)
Personnel	793 (17.8)	2,640 (24.5)	1,847 (29.2)	934 (16.2)	3,218 (22.3)	2,284 (26.3)
Transport	482 (10.8)	1,744 (16.2)	1,263 (19.9)	724 (12.6)	2,313 (16.0)	1,589 (18.3)
Supplies and services	62 (1.4)	384 (3.6)	322 (5.1)	67 (1.2)	397 (2.8)	330 (3.8)
**Drug distribution (opportunity)**	**86 (1.9)**	**52 (0.5)**	**-34 (-0.5)**	**110 (1.9)**	**57 (0.4)**	**-53 (-0.6)**
Medicines (ALB)	86 (1.9)	52 (0.5)	-34 (-0.5)	110 (1.9)	57 (0.4)	-53 (-0.6)
**Overheads (financial)**	**397 (8.9)**	**945 (8.8)**	**547 (8.6)**	**514 (8.9)**	**1,277 (8.8)**	**762 (8.8)**
**Total (financial and opportunity) costs**	**4,458 (100.0)**	**10,793 (100.0)**	**6,335 (100.0)**	**5,765 (100.0)**	**14,445 (100.0)**	**8,681 (100.0)**
Total financial costs	4,372 (98.1)	10,741 (99.5)	6,370 (100.5)	5,655 (98.1)	14,388 (99.6)	8,733 (100.6)
Total opportunity costs	86 (1.9)	52 (0.5)	-34 (-0.5)	110 (1.9)	57 (0.4)	-53 (-0.6)

*Note: Percentages (%) in parentheses were calculated relative to the total (financial and opportunity) costs for each arm or the total incremental (financial and opportunity) costs. A negative incremental cost or percentage indicates lower cost for a given activity or category in MDA-ALB-IVM compared with MDA-ALB.*

Across districts and arms, personnel costs represented the largest cost component (38.9-43.6%). When broken down by sub-categories, per diems were the largest cost component (22.2-30.3%), followed by service costs (15.7-20.0%) and allowances (13.3-16.8%). This is presented in Table H in S1 Text.

By activities, the major incremental costs were associated with drug distribution (including financial and opportunity costs) and training, accounting for 51.8-59.2% and 29.6-32.2% of the incremental costs, respectively. By cost categories, the main incremental costs were personnel (38.9-43.7%), transport (22.2-23.6%), and services and supplies (19.1-26.8%) (Table H in S1 Text).

### Treatment coverage and cost per child treated

In Kabale, the average treatment coverage and the number of SAC treated were lower in MDA-ALB-IVM compared with MDA-ALB: 56.2% (range 33.2-95.4%) versus 99.4% (range 97.2-100.0%) for the treatment coverage, and 1,354 versus 2,229 for the number of SAC treated (Table 2).

**Table 2:** Number of children treated (5-14 years) and treatment coverage (%).

District	Arm	Total number of eligible children (5–14 years)	Number of children treated	Average coverage (min-max)
Kabale	MDA-ALB	2,235	2,229	99.4 (97.2-100.0)
	MDA-ALB-IVM	2,666	1,354	56.2 (33.2- 95.4)
Kisoro	MDA-ALB	2,870	2,833	98.9 (98.0-100.0)
	MDA-ALB-IVM	1,568	1,476	94.1 (91.5- 97.7)

In Kisoro, where audio announcements were broadcast and VHTs alone or in collaboration with local community leaders were involved, the average treatment coverage was high in both arms (98.9% and 94.1% in MDA-ALB and MDA-ALB-IVM, respectively). The total number of SAC treated with ALB alone (2,833) was higher than the number of SAC treated with ALB-IVM (1,476).

The cost per child treated was calculated as the total (financial and opportunity) costs divided by the total number of eligible children treated. Higher costs and a lower number of children treated in MDA-ALB-IVM led to markedly higher cost per child treated in MDA-ALB-IVM compared with MDA-ALB (as presented in Table 3). The cost per child treated was $2.00-$2.03 and $7.97-$9.79 for MDA-ALB and MDA-ALB-IVM, respectively.

**Table 3:** Pilot-based cost per child treated (US$).

Activity/ cost category	Kabale		Kisoro	
	MDA-ALB	MDA-ALB-IVM	MDA-ALB	MDA-ALB-IVM
**Advocacy (financial)**	**0.19**	**0.31**	**0.15**	**0.29**
Personnel	0.09	0.15	0.07	0.14
Transport	0.10	0.17	0.08	0.15
**Training (financial)**	**0.99**	**3.14**	**0.98**	**3.62**
Personnel	0.42	1.37	0.39	1.50
Transport	0.14	0.40	0.15	0.52
Supplies and services	0.43	1.36	0.43	1.61
**Sensitization (financial)**	**0.00**	**0.00**	**0.08**	**0.72**
Supplies and services	0.00	0.00	0.08	0.72
**Drug distribution (financial)**	**0.60**	**3.78**	**0.61**	**4.25**
Medicines (IVM)	0.00	0.26	0.00	0.23
Personnel	0.36	1.95	0.33	2.18
Transport	0.22	1.29	0.26	1.57
Supplies and services	0.03	0.28	0.02	0.27
**Drug distribution (opportunity)**	**0.04**	**0.04**	**0.04**	**0.04**
Medicines (ALB)	0.04	0.04	0.04	0.04
**Overheads (financial)**	**0.18**	**0.70**	**0.18**	**0.86**
**Total (financial and opportunity) costs**	**2.00**	**7.97**	**2.03**	**9.79**
Total financial costs	1.96	7.93	2.00	9.75
Total opportunity costs	0.04	0.04	0.04	0.04

*Note: Cost per child treated was calculated as the pilot-based costs divided by the number of children (aged 5–14 years) treated.*

### Scenario analysis

Table 4 shows the scenario-adjusted costs. When the number of days for drug distribution and personnel requirements were reduced in MDA-ALB-IVM, the incremental (financial and opportunity) costs of MDA-ALB-IVM compared with MDA-ALB decreased by 31% and 36% in Kabale and Kisoro, respectively. The major incremental costs were associated with training (46.5%) and drug distribution (37.1-45.0%, including financial and opportunity costs). By cost categories, the incremental costs were primarily driven by personnel (28.5-29.1%), transport (23.3-27.8%) and supplies and services (27.5-34.3%) (Table O in S1 Text).

**Table 4:** Scenario-adjusted* cost (US$) comparison of MDA-ALB and MDA-ALB-IVM.

Activity/ cost category	Total costs (%)		Incremental costs (%)	Cost per child treated	
	MDA-ALB	MDA-ALB-IVM		MDA-ALB	MDA-ALB-IVM
Kabale district					
Advocacy (financial)	425 (9.6)	425 (4.8)	0 (0.0)	0.19	0.31
Training (financial)	2,212 (49.9)	4,255 (48.2)	2,042 (46.5)	0.99	3.14
Sensitization (financial)	0 (0.0)	0 (0.0)	0 (0.0)	0.00	0.00
Drug distribution (financial)	1,315 (29.7)	3,329 (37.7)	2,014 (45.8)	0.59	2.46
Drug distribution (opportunity)	86 (1.9)	52 (0.6)	-34 (-0.8)	0.04	0.04
Overheads (financial)	395 (8.9)	766 (8.7)	371 (8.4)	0.18	0.57
Total (financial and opportunity) costs	4,435 (100.0)	8,828 (100.0)	4,393 (100.0)	1.99	6.52
**Difference**^**‡**^ **(%) in total costs compared with the pilot-based costs**	**-1%**	**-18%**	**-31%**	**-1%**	**-18%**
Kisoro district					
Advocacy (financial)	425 (7.4)	425 (3.8)	0 (0.0)	0.15	0.29
Training (financial)	2,777 (48.3)	5,347 (47.4)	2,570 (46.5)	0.98	3.62
Sensitization (financial)	213 (3.7)	639 (5.7)	426 (7.7)	0.08	0.43
Drug distribution (financial)	1,712 (29.8)	3,821 (33.9)	2,077 (38.1)	0.60	2.59
Drug distribution (opportunity)	110 (1.9)	57 (0.5)	-53 (-1.0)	0.04	0.04
Overheads (financial)	513 (8.9)	989 (8.8)	476 (8.6)	0.18	0.67
Total (financial and opportunity) costs	5,750 (100.0)	11,277 (100.0)	5,496 (100.0)	2.03	7.64
**Difference**^**‡**^ **(%) in total costs compared with the pilot-based costs**	**~0%**	**-22%**	**-36%**	**0%**	**-22%**

Note: Percentages (%) in parentheses were calculated relative to the total (financial and opportunity) costs for each arm or the total incremental (financial and opportunity) costs. A negative incremental cost or percentage indicates lower cost for a given activity in MDA-ALB-IVM compared with MDA-ALB.

*The scenario assumed a reduced number of days and reduced personnel requirements for drug distribution in MDA-ALB-IVM in the absence of the informed consent and assent process, while the number of children treated remained unchanged.

^‡^Difference in % compared with the pilot-based costs was calculated as (total scenario-adjusted costs – total pilot-based costs)/ total pilot-based costs × 100%. The pilot-based costs are presented in Tables 1 and 3.

The estimated cost per child treated in the MDA-ALB-IVM scenario decreased to $6.52 (-18%) in Kabale and to $7.64 (-22%) in Kisoro. The costs and/or the estimated cost per child treated in MDA-ALB changed slightly due to scenario-adjusted cost re-allocation. Detailed scenario-adjusted costs by activities and/or cost categories are presented in Tables N-Q in S1 Text.

### Treatment time in schools

Table 5 presents the treatment time in schools and the average time per 100 children treated, which varied across districts and arms. The average time per 100 children treated was higher in MDA-ALB-IVM than in MDA-ALB.

**Table 5:** Treatment time in schools.

District	Arm	Number of children treated	Treatment time in schools		Average time per 100 children treated
			Mean	Min - max	
Kabale	MDA-ALB	2,229	2 h 14 min	0 h 43 min - 3 h 37 min	0 h 30 min 51 s
	MDA-ALB-IVM	1,354	4 h 30 min	1 h 40 min - 7 h 34 min	1 h 34 min 13 s
Kisoro	MDA-ALB	2,833	4 h 24 min	1 h 32 min - 8 h 43 min	0 h 47 min 9 s
	MDA-ALB-IVM	1,476	4 h 54 min	4 h 32 min - 5 h 26 min	2 h 9 min 26 s

Note: Treatment time was calculated as the difference between treatment start time and stop time in schools. For MDA-ALB-IVM, the treatment time included the time required for assenting (assent and treatment were conducted sequentially); children aged eight years and above received treatment immediately after assenting, and children aged under eight years received treatment when their name and class matched their signed parental consent forms. Time per 100 children treated = treatment time/ number of eligible children (5–14 years) treated * 100.

### Sensitivity analyses

**Fig 1:**
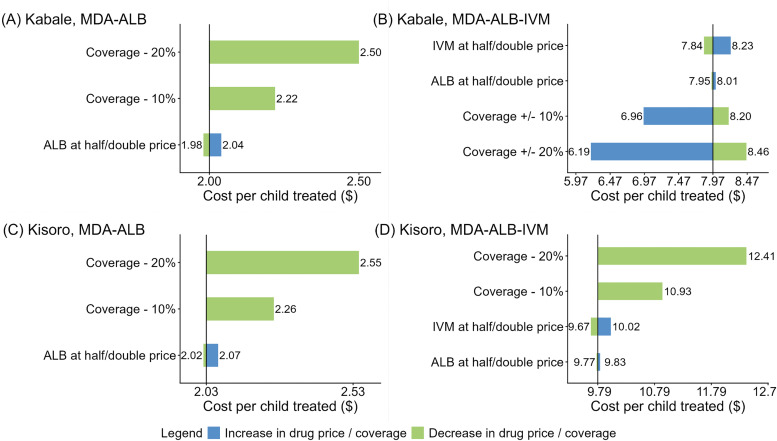
Tornado plots of sensitivity analyses. Note: Bars show the estimated pilot-based cost per child treated (aged 5-14 years) under various sensitivity scenarios in different intervention settings. The cost per child treated was calculated as the pilot-based total (financial and opportunity) costs divided by the number of eligible children (aged 5-14 years) treated. Bars in blue represent the estimated cost per child treated after increasing drug price or treatment coverage, while bars in green represent the estimated cost per child treated after decreasing drug price or treatment coverage. The central value in each panel reflects the base-case estimate without varying drug price or treatment coverage, while bars indicate how this estimate shifts under alternative assumptions for drug pricing and treatment coverage. Wider bars represent greater sensitivity to the parameter in question, highlighting which assumptions most influence the estimates of the cost per child treated. ALB, albendazole; IVM, ivermectin.

Varying the drug prices made small differences in the estimated cost per child treated, which was more sensitive to the treatment coverage (Fig 1 and Table R in S1 Text). For example, a 10% and 20% decrease in coverage resulted in cost per child treated increasing to $2.22 (+11%) and $2.50 (+25%) for MDA-ALB in Kabale, and $2.26 (+11%) and $2.55 (+26%) for MDA-ALB in Kisoro, respectively. When the coverage of MDA-ALB-IVM increased by 10% and 20% for schools with coverage <50% in Kabale, the cost per child treated decreased to $6.96 (-13%) and $6.19 (-22%), respectively.

## Discussion

This study assessed the costs of school-based MDA-ALB-IVM compared with MDA-ALB in a small-scale implementation pilot. The results show that the total (financial and opportunity) costs of ALB-IVM co-administration were higher than ALB alone, with personnel costs being the largest cost component. The main cost drivers for both total and incremental costs were training and drug distribution. Incremental costs were greatly influenced by the number of days used for drug distribution, while the cost per child treated was sensitive to variations in costs (especially due to number of days for drug distribution) and number of children treated.

This study was not designed to compare the estimated cost per child treated to those reported in larger-scale studies [12,14,15,28,29], as it was implemented at a limited scale of 5 schools with around 2’500 SAC targeted per arm per district. Previous research has shown that MDA programs benefit from economies of scale, with the cost per person treated decreasing as the number of persons treated increases [15,16].

Given that IVM was purchased in this study, the costs of IVM were categorized as financial costs, while ALB was donated and, therefore, the costs of ALB were categorized as opportunity costs. Drugs costs accounted for small shares of the total costs (1.9% in MDA-ALB and 2.8-3.7% in MDA-ALB-IVM; Table H in S1 Text).

The study results align with published literature that personnel costs were the primary cost driver [12,14,17,28], with training [14,15,17] and drug distribution [12,14,15,28] substantially contributing to the total costs of MDA. However, a few studies also reported community sensitization as a key contributor to the financial and economic costs [12,17,28]. In addition, training costs were found to contribute significantly less to the total costs when compared with community sensitization and drug administration [12,28].

In each district, teachers and health workers from both arms were invited to one training, of which the content included the usage of the dose pole to determine the number of IVM tablets to be administered. According to the experiences of routine deworming campaigns in Uganda and as advised by MoH, a one-day training is sufficient for MDA-ALB. Therefore, when allocating training costs to each arm, one-day training costs were allocated to MDA-ALB, while the full two-day training costs were allocated to MDA-ALB-IVM. This explains 29.6-32.2% of the pilot-based incremental costs; 46.5% of scenario-adjusted incremental costs were associated with training.

Given the experimental nature of the implementation pilot and that ALB-IVM was a new intervention for STH infections in Uganda, individual informed consent and assent procedures were required (by the ethical authorities as the implementation pilot was seen as a research project) for MDA-ALB-IVM. Therefore, more days and personnel were required for drug distribution of ALB-IVM relative to ALB alone. This led to considerable incremental costs of MDA-ALB-IVM compared with MDA-ALB. When assuming a reduced number of days and lower personnel requirements for drug distribution in MDA-ALB-IVM without an informed consent and assent process, as illustrated by the scenario analysis, the total incremental costs decreased by 31% in Kabale and 36% in Kisoro.

The actual drug administration procedures of IVM differed from the standard ALB distribution, which was provided as a single tablet of 400 mg. Each child in MDA-ALB-IVM had to be measured with a dose pole and the correct number of IVM tablets had to be administered as indicated on the pole [19]. This increased time requirement for the ALB-IVM co-administration is reflected by the higher treatment time per 100 children for MDA-ALB-IVM compared with that for MDA-ALB. Of note, a fixed-dose, orodispersible tablet that combines ivermectin and albendazole is currently under development that would allow a more efficient treatment [30].

Additionally, when examining the costs of MDA across districts, we observed higher costs in Kisoro than in Kabale. This reflects the greater use of resources in Kisoro, such as more personnel (VHTs and local community leaders) involved in drug distribution and audio announcements for intensified community sensitization. Once MDA-ALB-IVM becomes routine, the need for intensified community sensitization is likely to decrease, as well as intensified supervision from MoH.

The observed low treatment coverage of MDA-ALB-IVM in Kabale (due to refusal to provide informed consent and/or assent) reflects the importance of adequate community sensitization and the involvement of VHTs and local community leaders. MDA-ALB is the routine for school-based MDA in Uganda, while MDA-ALB-IVM is a new treatment intervention for STH infections. Of note, IVM is already used to treat onchocerciasis [31–33] and ALB-IVM co-administration is used for lymphatic filariasis treatment in Uganda [33,34]. In both districts, there were some concerns and negative perceptions that the medicines might be harmful in the frame of the STH program. The negative perceptions were primarily linked to the informed consent and assent procedures, which is not required in routine deworming campaigns and raised community mistrust about why they would need to sign a form for a government-led MDA campaign and allegedly safe treatment. In Kabale, there was no radio announcement about the MDA campaign prior to treatment (unlike the routine government-led deworming campaigns in Uganda). Furthermore, VHTs or local community leaders were not involved during the drug distribution. Such conditions might have increased the communities’ concerns about the safety of the treatment, which may have led to refusal to consent and/or assent and thus low coverage for MDA-ALB-IVM. On the contrary, in Kisoro, there were radio announcements prior to and during drug distribution days to sensitize and mobilize SAC and communities, and VHTs alone or in collaboration with local community leaders were involved during the drug distribution. The treatment coverage of MDA-ALB-IVM in Kisoro was similar to that of MDA-ALB.

The number of children treated with ALB-IVM was significantly lower than the number treated with ALB alone in both districts, but attributable to different factors. In Kabale, the lower number of children treated was primarily due to lower treatment coverage in MDA-ALB-IVM. However, in Kisoro, the treatment coverage was similarly high (>90%) in both arms, and the lower number of children treated with ALB-IVM was mainly the result of a lower number of eligible children in MDA-ALB-IVM assigned schools. The difference in the eligible population size may be explained by several factors: some enrolled children may have been ineligible (aged below 5 years and 15 years and above, Table K in S1 Text); there may have been drop-outs after enrollment; or pre-randomization estimates of school enrollment sizes may have been over- or underestimated. The differences in the number of children treated between arms warrant caution when comparing the cost per child treated, particularly if the implementation scale remains small.

In both districts, the costs of MDA-ALB-IVM were higher than those of MDA-ALB and the number of children treated was lower in MDA-ALB-IVM than in MDA-ALB. These led to a substantially higher cost per child treated with ALB-IVM than the cost per child with ALB alone. Similarly, in the scenario analysis, the cost per child treated for MDA-ALB-IVM decreased, though it remained higher than that in MDA-ALB. If the number of children treated in MDA-ALB-IVM increased to the same number as in MDA-ALB (2,229 in Kabale and 2,833 in Kisoro), the scenario-adjusted cost per child treated with ALB-IVM would decrease to $4.07 (by 49%) in Kabale and $4.11 (by 58%) in Kisoro (Table M in S1 Text), around twice the scenario-adjusted cost per child treated with ALB alone.

There are opportunities to integrate MDA-ALB-IVM into the existing Integrated Child Health Days (ICHDs) in Uganda, where MDA with ALB alone has already been integrated. ICHDs are led by MoH with the aim to improve health outcomes with particular focus on child and maternal health [35]. ICHDs usually take place in April and October, providing health services such as immunizations, vitamin A supplementation, and deworming with ALB or mebendazole. Integrating MDA-ALB-IVM into ICHDs could decrease MDA implementation costs, benefiting from synergies in community sensitization and mobilization, as well as economies of scope (the reduction in cost per child treated when more than one intervention is delivered at once). However, additional training may be necessary for determining the correct number of IVM tablets using a dose pole, though the two-day training could be reduced to a one-day training when the combination becomes a routine treatment for STH infection control.

This study has some limitations. Firstly, as mentioned above, this study was implemented at a relatively limited scale. Therefore, comparisons of the cost per child treated with estimates from larger-scale studies should be made with caution, as they may not account for potential economies of scale. Secondly, this study was implemented as a pilot and could still be considered experimental rather than mirroring real routine processes due to the requirement to obtain individual consent and assent in MDA-ALB-IVM and the need of intensified community sensitization to explain non-standard procedures. Thirdly, although schools were randomly selected from a list of schools with an enrollment of about 500 pupils, the marked difference in the total number of eligible children between arms in Kisoro limits the validity of direct comparisons of cost per child treated between the two interventions. Additionally, the recorded treatment time in schools of MDA-ALB-IVM included the time required for child assent. Furthermore, the variations in resource inputs also warrant caution when comparing the total costs and the cost per child treated across districts and interventions. Lastly, the opportunity costs of personnel time were not estimated because data on personnel salaries were unavailable, which would have increased the personnel costs and the incremental costs. Future studies could conduct a salary survey to collect these data and allow for the estimation of the opportunity costs of personnel time and, thus, a more complete evaluation of the total economic costs of MDA implementation.

Despite these limitations, this is, to our knowledge, the first study to compare the total financial and opportunity costs (of donated ALB) of school-based MDA-ALB-IVM versus MDA-ALB for STH control. The study identified key cost drivers and found that adequate community sensitization, together with the involvement of community health workers (e.g., VHTs in Uganda) and local community leaders, is critical to achieving high treatment coverage, especially at the initial rollout of a new intervention. The findings of this study provide real-world evidence from the introduction of MDA-ALB-IVM in Uganda and offer practical guidance for the broader implementation of ALB-IVM co-administration.

## Supporting information

S1 Text Supporting tables for method description and results and tornado plots of scenario-adjusted sensitivity analyses. Table A Mass drug administration (MDA) activities, description and resources used. Table B Cross-district and cross-arm activity organization and staffing. Table C Personnel involvement and roles, number of activity days and the associated costs. Table D Cost categories and sub-categories. Table E Allocation approach for shared costs. Table F Scenario-adjusted number of drug distribution days and personnel required per school. Table G Unit price/cost for some key items. Table H Pilot-based total and incremental costs (US$) by cost categories and sub-categories. Table I Number of children treated (5–14 years) and treatment coverage (%). Table J Treatment time in schools (all ages). Table K Number of children treated and treatment coverage per school. Table L Pilot-based cost per child treated (US$) by cost categories and sub-categories. Table M Scenario analysis (difference in % compared with the pilot-based costs). Table N Scenario-adjusted total and incremental costs (US$) by activities and cost categories. Table O Scenario-adjusted total and incremental costs (US$) by cost categories and sub-categories. Table P Scenario-adjusted cost per child treated (US$) by activities and cost categories. Table Q Scenario-adjusted cost per child treated (US$) by cost categories and sub-categories. Table R Sensitivity analyses (difference in % compared with the pilot-based cost per child treated). Figure A Tornado plots of scenario-adjusted sensitivity analyses.
(DOCX)
