# Supplementary material for: Cost comparison of school-based mass drug administration of albendazole and ivermectin versus albendazole alone for soil-transmitted helminth control in Uganda
Source: PLoS Negl Trop Dis. 2026 Jan 14;20(1):e0013913. doi: 10.1371/journal.pntd.0013913 (PMC12829928; doi:10.1371/journal.pntd.0013913)
Supplement: S1 Text — Table A Mass drug administration (MDA) activities, description and resources used. Table B Cross-district and cross-arm activity organization and staffing. Table C Personnel involvement and roles, number of activity days and the associated costs. Table D Cost categories and sub-categories. Table E Allocation approach for shared costs. Table F Scenario-adjusted number of drug distribution days and personnel required per school. Table G Unit price/cost for some key items. Table H Pilot-based total and incremental costs (US$) by cost categories and sub-categories. Table I Number of children treated (5–14 years) and treatment coverage (%). Table J Treatment time in schools (all ages). Table K Number of children treated and treatment coverage per school. Table L Pilot-based cost per child treated (US$) by cost categories and sub-categories. Table M Scenario analysis (difference in % compared with the pilot-based costs). Table N Scenario-adjusted total and incremental costs (US$) by activities and cost categories. Table O Scenario-adjusted total and incremental costs (US$) by cost categories and sub-categories. Table P Scenario-adjusted cost per child treated (US$) by activities and cost categories. Table Q Scenario-adjusted cost per child treated (US$) by cost categories and sub-categories. Table R Sensitivity analyses (difference in % compared with the pilot-based cost per child treated). Figure A Tornado plots of scenario-adjusted sensitivity analyses. (DOCX) [file pntd.0013913.s001.docx]

**S1 Text**

**Supporting tables for method description and results and tornado plots of scenario-adjusted sensitivity analyses**

Cost comparison of school-based mass drug administration of albendazole and ivermectin versus albendazole alone for soil-transmitted helminth control in Uganda

Yuling Lin^1,2^*, Tanja Barth-Jaeggi^1,2^, Eveline Hürlimann^1,2^, Prudence Beinamaryo^3^, Hilda Kyarisiima^3^, Harsh Vivek Harkare^1,2^, Leonsio Matagi^4^, Isaac Byarugaba^4^, Peter Steinmann^1,2^, Jennifer Keiser^1,2^, Fabrizio Tediosi^1,2^

1. Swiss Tropical and Public Health Institute, Allschwil, Switzerland
2. University of Basel, Basel, Switzerland
3. Vector Borne and Neglected Tropical Diseases Division, Ministry of Health, Kampala, Uganda
4. College of Humanities and Social Sciences, Makerere University, Kampala, Uganda

* Corresponding author: yuling.lin@swisstph.ch

Contents

[**Table A** Mass drug administration (MDA) activities, description and resources used 2](#_Toc218592277)

[**Table B** Cross-district and cross-arm activity organization and staffing 3](#_Toc218592278)

[**Table C** Personnel involvement and roles, number of activity days and the associated costs 4](#_Toc218592279)

[**Table D** Cost categories and sub-categories 7](#_Toc218592280)

[**Table E** Allocation approach for shared costs 7](#_Toc218592281)

[**Table F** Scenario-adjusted number of drug distribution days and personnel required per school 8](#_Toc218592282)

[**Table G** Unit price/cost for key items 9](#_Toc218592283)

[**Table H** Pilot-based total and incremental costs ($) by cost categories and sub-categories 10](#_Toc218592284)

[**Table I** Number of children treated (5-14 years) and treatment coverage (%) 11](#_Toc218592285)

[**Table J** Treatment time in schools (all ages) 11](#_Toc218592286)

[**Table K** Number of children treated and treatment coverage per school 12](#_Toc218592287)

[**Table L** Pilot-based cost per child treated ($) by cost categories and sub-categories 13](#_Toc218592288)

[**Table M** Scenario analysis (difference in % compared with the pilot-based costs) 14](#_Toc218592289)

[**Table N** Scenario-adjusted total and incremental costs ($) by activities and cost categories 15](#_Toc218592290)

[**Table O** Scenario-adjusted total and incremental costs ($) by cost categories and sub-categories 16](#_Toc218592291)

[**Table P** Scenario-adjusted cost per child treated ($) by activities and cost categories 17](#_Toc218592292)

[**Table Q** Scenario-adjusted cost per child treated ($) by cost categories and sub-categories 18](#_Toc218592293)

[**Table R** Sensitivity analyses (difference in % compared with the pilot-based cost per child treated) 19](#_Toc218592294)

[**Figure A** Tornado plots of scenario-adjusted sensitivity analyses 20](#_Toc218592295)

# **Table A** Mass drug administration (MDA) activities, description and resources used

| **Activity** | **Description** | **Resources used** | **Note** |
| --- | --- | --- | --- |
| Advocacy | Prior to the treatment interventions, an advocacy meeting was held between a team from the Uganda Ministry of Health (MoH) and districts’ local governments to discuss about districts’ preparedness for MDA activities. | Per diems, car hiring, fuel |  |
| Training | In each district, the MoH staff (central supervisors) provided training to district health workers and schoolteachers on different aspects about the diseases, treatment and documentation procedures to be applied. Before training, district neglected tropical disease (NTD) focal persons^[[1]](#footnote-1)^ did mobilization and preparations for the training. In Kisoro, the Assistant District Health Officer, the District Education Officer and village health team (VHT)^[[2]](#footnote-2)^ coordinators were also invited to the training. | Allowances, transport refunds, hotel packages (including morning teas and lunches), projector renting, per diems for trainers, car hiring, fuel, airtime, stationery | The training content included disease epidemiology, transmission and control strategies, drug dosing and administration, adverse event reporting and administration, and treatment documentation and drug accountability using tally sheets, etc. |
| Community sensitization | In Kabale, there was no radio announcement about the MDA campaign prior distribution or involvement of VHTs.  In Kisoro, radio announcements about the MDA campaign were broadcast prior to and during the drug distribution days to sensitize communities.  In both districts, teachers sensitized children and communities about the MDA campaigns and mobilized children to receive treatment. Additionally in Kisoro, VHTs supported to mobilize communities for both arms. | Radio announcements |  |
| Drug distribution | Heath workers visited schools and administered medicines to schoolchildren with teachers assisting in organizational aspects. Central supervisors oversaw the whole process and/or supported wherever needed during drug distribution. In Kisoro, in addition to schoolteachers, VHTs were engaged in both arms, while local community leaders also supported in MDA-ALB-IVM.  For MDA-ALB, children, who were absent on the drug distribution day, were treated with tablets left to teachers who observed them swallow tablets when absent children were present in schools. Treatment follow-up was conducted by district focal persons, either through phone calls or visits to schools, to confirm whether the left tablets were taken by those absent children. Additionally, there was one school of MDA-ALB in Kisoro, where the prepared tablets were not enough to treat an unexpectedly large number (>1000) of children in that school. Those untreated children were treated during a follow-up visit by central supervisors.  For MDA-ALB-IVM, there was no follow-up visit for those who were not treated on the drug distribution days, except at one school in Kabale, where there was high reluctance, a follow-up visit was conducted, and some more children were treated. | Albendazole (ALB), ivermectin (IVM), allowances, per diems, transport refunds, stationery, dose poles, car hiring, fuel, drinking water for swallowing medicines, disposable cups and spoons, printing | ALB was obtained from the WHO (donated by GlaxoSmithKline). IVM (generic) was purchased from Laboratorios Liconsa, Spain.  Children aged 5-14 years were eligible to participate in this pilot and were treated with the respective medicines depending on which arm the schools were assigned to. In order not to leave anyone behind, children under 5 years received the routine MDA intervention (ALB alone), whereas the treatment for children aged 15 years and above depended on the drug distribution teams in districts. In Kabale, children aged 15+ years received ALB-IVM, while in Kisoro, they received ALB alone. |

*Abbreviations: NTD, neglected tropical disease; MDA, mass drug administration; VHT, village health team; MDA-ALB, mass drug administration with albendazole alone; MDA-ALB-IVM, mass drug administration with the combination of albendazole and ivermectin.*

# **Table B** Cross-district and cross-arm activity organization and staffing

| **Activity / personnel** | **Kabale** | | **Kisoro** | |
| --- | --- | --- | --- | --- |
|  | **MDA-ALB** | **MDA-ALB-IVM** | **MDA-ALB** | **MDA-ALB-IVM** |
| Training | Participants: district health workers and schoolteachers  Trainers: central supervisors and district neglected tropical disease (NTD) focal person | | Participants: district health workers, schoolteachers and VHT coordinators; assistant District Health Officer and District Education Officer were also invited.  Trainers: central supervisors and district NTD focal person | |
| Personnel deployment for drug distribution | - Three health workers - Four MoH staff - One NTD focal person - On average three schoolteachers per school | | - Five health workers - Five MoH staff - One NTD focal person - On average three schoolteachers per school - On average one VHT per school | - Five health workers - Five MoH staff - One NTD focal person - On average three schoolteachers per school - On average two to three VHTs and one to two local community leaders per school |
| Community sensitization | No audio announcement, no involvement of VHTs | | With audio announcement prior and/or during drug distribution days, with involvement of VHTs and local community leaders | |
| Drug distribution (arrangement of personnel and number of drug distribution days) | **Arrangement of personnel**: the drug distribution team was arranged into 5 groups to treat 5 schools in one day  **Number of days for drug distribution**: 1 day | **Arrangement of personnel**: the whole drug distribution team treated one school per day  **Number of days for drug distribution**: 5 days | **Arrangement of personnel**: the drug distribution team was arranged into 5 groups to treat 5 schools in one day  **Number of days for drug distribution**: 1 day | **Arrangement of personnel**: the whole drug distribution team treated children in one school per day  **Number of days for drug distribution**: 5 days |

*Abbreviations: NTD, neglected tropical disease; MDA, mass drug administration; VHT, village health team; MDA-ALB, mass drug administration with albendazole alone; MDA-ALB-IVM, mass drug administration with the combination of albendazole and ivermectin.*

# **Table C** Personnel involvement and roles, number of activity days and the associated costs

| **Activity** | **Personnel involved** | **Personnel role** | **Number of activity days** | **Associated financial and opportunity costs that were included in the analysis** |
| --- | --- | --- | --- | --- |
| Advocacy | Central supervisors | Central supervisors: informed the district governments about the MDA activities and discussed with district governments about districts’ preparedness for MDA activities. | 1 | Financial costs: car hire, fuel, per diems (central supervisors) |
|  | District governments: E.g., District Health Officer, Assistant District Health Officer, District NTD focal person, District Education Officer | Discussed about districts’ preparedness for MDA activities with central supervisors. |  |  |
| Training | Central supervisors | Trainers at the training | 2 | Financial costs: per diems (central supervisors), allowances and transport refund (district NTD focal person, health workers, schoolteachers, VHT coordinators, Assistant District Health Officer, and District Education Officer), hotel packages (including morning teas and lunches), projector renting, car hiring, fuel, airtime, stationery |
|  | District NTD focal persons | Mobilized training participants and supported and facilitated in the training wherever needed. |  |  |
|  | Health workers, schoolteachers | Training participants |  |  |
|  | VHT coordinators | Training participants in Kisoro only. These trained VHT coordinators later informed and engaged another 1 or 2 VHT members and community leaders from villages of catchment areas. |  |  |
|  | Assistant District Health Officer, District Education Officer | Training participants in Kisoro |  |  |
| Community sensitization | Schoolteachers | At both districts, schoolteachers announced the deworming to and sensitize pupils at schools and/or churches (community members would also be sensitized when the announcement was done at churches) and mobilized pupils to come to schools on the day of treatment. | 1-6 days (not explicitly documented in the pilot). At least one day, depending on schoolteachers, VHTs and community leaders.  Audio announcements were running for 6 days in Kisoro. | Financial costs: audio announcements |
|  | VHTs | Sensitized and mobilized pupils and community members in villages in both MDA-ALB and MDA-ALB-IVM. |  |  |
|  | Community leaders | Supported mobilization in MDA-ALB-IVM, Kisoro district. |  |  |
| Drug distribution | Health workers | Administered medicines to schoolchildren | 1 day for MDA-ALB, and 5 days for MDA-ALB-IVM | Financial costs: per diems (central supervisors), allowances (health workers, schoolteachers, District NTD focal persons, VHTs and community leaders), transport refunds (District NTD focal persons, VHTs), IVM, stationery, dose poles, car hiring, fuel, drinking water for swallowing medicines, disposable cups and spoons, printing  Opportunity costs: ALB |
|  | Schoolteachers | Assisted in documentation and organizational aspects, such as dose dtermination using a dose pole for IVM, children/class arrangement and observing tablets swallowing. |  |  |
|  | Central supervisors | Oversaw the entire process and/or supported wherever needed during drug distribution. |  |  |
|  | District NTD focal persons | District facilitators, who facilitated and supported wherever needed during drug distribution. |  |  |
|  | VHTs | Supported in sensitization and mobilization in both MDA-ALB and MDA-ALB-IVM in Kisoro. |  |  |
|  | Community leaders | Supported in mobilization in MDA-ALB-IVM in Kisoro. |  |  |

*Note: 1) Number of days presented in the table does not include travel days; 2) Some activities, such as advocacy and community sensitization might not be a full-day activity, but only the number of days was documented instead of in hours; 3) Treatment time in schools were documented in hours and presented in Table 5 in the main text; 4) The opportunity costs of personnel time spent by all personnel on all MDA activities were not valued or included in the analysis as data on salary estimates were not collected in the pilot.*

*Abbreviations: NTD, neglected tropical disease; MDA, mass drug administration; VHT, village health team; MDA-ALB, mass drug administration with albendazole alone; MDA-ALB-IVM, mass drug administration with the combination of albendazole and ivermectin.*

# **Table D** Cost categories and sub-categories

| **Type of costs** | **Cost category** | **Cost sub-category** | **Item** |
| --- | --- | --- | --- |
| Financial costs | Medicines | Medicines | Ivermectin |
|  | Personnel | Allowances | Allowances (given to personnel other than central supervisors) |
|  |  | Per diems | Per diems (given to central supervisors) |
|  | Transport | Transport refund | Transport refund |
|  |  | Car hiring | Car hiring |
|  |  | Fuel | Fuel from Kampala to Kabale, fuel from Kabale to Kisoro, fuel from Kisoro to Kampala, fuel used in districts |
|  | Supplies and services | Services | Hotel packages (i.e., catering, including morning teas and lunches), projector renting, airtime |
|  |  | Stationery | Notebooks and pens, cartridge for the printer, other stationery (stappling machines, punching machines, stapples, masking tapes, markers, box files and ink pads, papers, paper clips) |
|  |  | Radio announcements | Radio announcements |
|  |  | Dose poles | Dose poles |
|  |  | Other supplies | Drinking water for swallowing medicines, disposable cups, disposable spoons, printing forms and sheets |
|  | Overheads | Overheads | Overheads (program administration and running costs, 10% of financial costs excluding the costs of ivermectin) |
| Opportunity costs | Medicines | Medicines | Albendazole |

# **Table E** Allocation approach for shared costs

| **Shared costs** | **Allocation approach** |
| --- | --- |
| Advocacy | 0.5:0.5 to each district and each arm |
| Fuel cost for travel to and from districts | 0.5:0.5 to each arm |
| Allowances, fuel and airtime for NTD focal persons for the mobilization and preparations for trainings | 0.5:0.5 to each arm |
| Airtime for trainers for trainings | 0.5:0.5 to each arm |
| Training costs | By number of activity days: 1-day training for the ALB arm while two-day training for the ALB-IVM, based on the experience that one-day training was sufficient for the ALB arm and two days were required for the ALB-IVM arm. |
| Notebooks and pens (training) | 0.5:0.5 to each arm |
| Printing | 0.5:0.5 to each arm |
| Stationery | Allocation to activities: by number of activity days; allocation to each district and each arm: 0.5:0.5 |
| Radio announcements | By number of drug distribution days |

*Abbreviations: NTD, neglected tropical disease; ALB, albendazole; ALB-IVM, combination of albendazole and ivermectin.*

# **Table F** Scenario-adjusted number of drug distribution days and personnel required per school

|  | **MDA-ALB (no change)** | **MDA-ALB-IVM (reduced n of days and personnel)** |
| --- | --- | --- |
| Kabale | | |
| N of days for drug distribution | One day  (all five schools in one day) | Three days (one or two schools per day), instead of five days (one school per day) |
| Personnel required per school | - One health worker - On average three teachers - One central supervisor - (or + one district NTD focal person) | - Two health workers, instead of three - On average three teachers - Two central supervisors, instead of four - One district NTD focal person |
| Kisoro | | |
| N of days for drug distribution | One day  (all five schools in one day) | Three days (one or two schools per day), instead of five days (one school per day) |
| Personnel required per school | - One health worker - On average three teachers - One central supervisor - One VHT - (or + one district NTD focal person) | - Two health workers, instead of four to five - On average three teachers - Two central supervisors, instead of three to five - Two to three VHTs - One to two local community leaders - One district NTD focal person |

*Note: To estimate the costs in the absence of informed consent and assent process, we explore one scenario: we assumed that in the absence of informed consent and assent process, the drug distribution team with reduced personnel treated SAC in one to two schools per day (three days for five schools) in MDA-ALB-IVM, and the organization in MDA-ALB remained unchanged – treating SAC in all five schools in one day. This scenario assumed a reduced number of days for drug distribution and reduced personnel requirements in MDA-ALB-IVM, while assuming the number of children treated remained unchanged.*

*Abbreviations: NTD, neglected tropical disease; MDA, mass drug administration; VHT, village health team; MDA-ALB, mass drug administration with albendazole alone; MDA-ALB-IVM, mass drug administration with the combination of albendazole and ivermectin.*

# **Table G** Unit price/cost for key items

| **Item** | **Unit price (UGX)** | **Unit price (USD, $)** | **Unit** | **Note** |
| --- | --- | --- | --- | --- |
| Albendazole | - | 0.0385 | Per tablet | $0.035 per tablet plus additional 10% for shipping costs. The price of $0.035 per tablet was inquired from the International Dispensary Association Foundation by personal communication via email in the year 2025. We assumed the 2024 price was the same as the 2025 price. |
| Ivermectin | - | 0.117172 | Per tablet | $0.10652 per tablet plus additional 10% for shipping costs. The price of $0.01065 per tablet was the price at which IVM purchased for this study in 2024, which is the same price at which WHO would pay for purchasing generic IVM from Laboratorios Liconsa, Spain.  The transport cost of IVM from Madrid, Spain to Basel, Switzerland was around 2.7% of the payment for IVM. Since IVM was brought to Uganda by researchers, i.e., no additional transport costs for transporting IVM from Basel to Uganda, and to try to not (or not too much) underestimate the shipping costs for IVM, we decided to add additional 10% (the same rate as for ALB shipping costs) of the IVM price to account for shipping costs. |
| Allowances | 50,000-160,000 | 13.31- 42.58 | Per person per day |  |
| Per diems | 300,000 | 79.84 | Per person per day |  |
| Transport refund | 10,000-50,000 | 2.66-13.31 | Per person per day |  |
| Car hiring | 300,000 | 79.84 | Per car per day |  |
| Fuel | 5,750 | 1.53 | Per liter |  |
| Hotel package | 120,000 | 31.94 | Per person per day | Including morning tea and lunch |
| Projector renting | 250,000 | 66.54 | Per day |  |
| Radio announcements | 40,000 | 10.65 | Per announcement |  |
| Dose pole | 100,000 | 26.62 | Per piece |  |

*Abbreviations: UGX, Ugandan schilling; USD, United State dollar.*

# **Table H** Pilot-based total and incremental costs ($) by cost categories and sub-categories

| **Cost category / sub-category** | **Kabale** | | | **Kisoro** | | |
| --- | --- | --- | --- | --- | --- | --- |
|  | **Total costs** | | **Incremental costs (%)** | **Total costs** | | **Incremental costs (%)** |
|  | **MDA-ALB** | **MDA-ALB-IVM** |  | **MDA-ALB** | **MDA-ALB-IVM** |  |
| **Medicines (financial)** | **0 (0.0)** | **348 (3.2)** | **348 (5.5)** | **0 (0.0)** | **346 (2.4)** | **346 (4.0)** |
| Ivermectin | 0 (0.0) | 348 (3.2) | 348 (5.5) | 0 (0.0) | 346 (2.4) | 346 (4.0) |
| **Medicines (opportunity)** | **86 (1.9)** | **52 (0.5)** | **-34 (-0.5)** | **110 (1.9)** | **57 (0.4)** | **-53 (-0.6)** |
| Albendazole | 86 (1.9) | 52 (0.5) | -34 (-0.5) | 110 (1.9) | 57 (0.4) | -53 (-0.6) |
| **Personnel (financial)** | **1,934 (43.4)** | **4,702 (43.6)** | **2,768 (43.7)** | **2,248 (39.0)** | **5,625 (38.9)** | **3,377 (38.9)** |
| Allowances | 648 (14.5) | 1,436 (13.3) | 788 (12.4) | 970 (16.8) | 2,152 (14.9) | 1,182 (13.6) |
| Per diems | 1,286 (28.8) | 3,266 (30.3) | 1,980 (31.3) | 1,278 (22.2) | 3,473 (24.0) | 2,196 (25.3) |
| **Transport (financial)** | **1,019 (22.8)** | **2,516 (23.3)** | **1,498 (23.6)** | **1,383 (24.0)** | **3,307 (22.9)** | **1,923 (22.2)** |
| Car hiring | 483 (10.8) | 1,367 (12.7) | 884 (13.9) | 552 (9.6) | 1,643 (11.4) | 1,091 (12.6) |
| Fuel | 461 (10.3) | 974 (9.0) | 513 (8.1) | 679 (11.8) | 1,312 (9.1) | 632 (7.3) |
| Transport refund | 75 (1.7) | 176 (1.6) | 101 (1.6) | 152 (2.6) | 351 (2.4) | 200 (2.3) |
| **Supplies and services (financial)** | **1,022 (22.9)** | **2,231 (20.7)** | **1,209 (19.1)** | **1,510 (26.2)** | **3,834 (26.5)** | **2,324 (26.8)** |
| Services | 892 (20.0) | 1,757 (16.3) | 865 (13.7) | 1,152 (20.0) | 2,268 (15.7) | 1,115 (12.8) |
| Stationery | 129 (2.9) | 150 (1.4) | 22 (0.3) | 143 (2.5) | 164 (1.1) | 22 (0.3) |
| Radio announcements | 0 (0.0) | 0 (0.0) | 0 (0.0) | 213 (3.7) | 1,065 (7.4) | 852 (9.8) |
| Dose poles | 0 (0.0) | 80 (0.7) | 80 (1.3) | 0 (0.0) | 80 (0.6) | 80 (0.9) |
| Other supplies | 2 (0.0) | 244 (2.3) | 242 (3.8) | 2 (0.0) | 257 (1.8) | 256 (2.9) |
| **Overheads (financial)** | **397 (8.9)** | **945 (8.8)** | **547 (8.6)** | **514 (8.9)** | **1,277 (8.8)** | **762 (8.8)** |
| **Total (financial and opportunity) costs** | **4,458 (100.0)** | **10,793 (100.0)** | **6,335 (100.0)** | **5,765 (100.0)** | **14,445 (100.0)** | **8,681 (100.0)** |
| Total financial costs | 4,372 (98.1) | 10,741 (99.5) | 6,370 (100.5) | 5,655 (98.1) | 14,388 (99.6) | 8,733 (100.6) |
| Total opportunity costs | 86 (1.9) | 52 (0.5) | -34 (-0.5) | 110 (1.9) | 57 (0.4) | -53 (-0.6) |

*Note: Percentages (%) in parentheses were calculated relative to the total (financial and opportunity) costs for each arm or the total incremental (financial and opportunity) costs. A negative incremental cost or percentage indicates lower cost for a given category or sub-category in MDA-ALB-IVM compared with MDA-ALB. Services included hotel packages (catering), projector renting, and airtime (Table D Cost categories and sub-categories).*

*Abbreviations: MDA-ALB, mass drug administration with albendazole alone; MDA-ALB-IVM, mass drug administration with the combination of albendazole and ivermectin.*

# **Table I** Number of children treated (5-14 years) and treatment coverage (%)

| **District** | **Arm** | **Total number of eligible children** | **Before follow-up** | | **After follow-up*** | |
| --- | --- | --- | --- | --- | --- | --- |
|  |  |  | **Number of treated** | **Average coverage (min-max)** | **Number of treated** | **Average coverage (min-max)** |
| Kabale | MDA-ALB | 2,235 | 2,150 | 93.1 (72.7-100.0) | 2,229 | 99.4 (97.2-100.0) |
|  | MDA-ALB-IVM | 2,666 | 1,323 | 55.3 (33.2-95.4) | 1,354 | 56.2 (33.2-95.4) |
| Kisoro | MDA-ALB | 2,870 | 2,652 | 93 (87.0-100.0) | 2,833 | 98.9 (98.0-100.0) |
|  | MDA-ALB-IVM | 1,568 | 1,476 | 94.1 (91.5-97.7) | 1,476 | 94.1 (91.5-97.7) |

*Note: *For MDA-ALB, there was follow-up either through phone calls or visits to schools, to confirm whether the left tablets were taken by those absent children or to treat untreated children on an occasion where not enough tablets were prepared on the scheduled drug distribution day. For MDA-ALB-IVM, there was no follow-up visit for those who were not treated on the scheduled drug distribution days, except at one school in Kabale, where there was high reluctance, a follow-up visit was conducted, and some more children were treated. That’s why as presented in the table, the number of treated and average coverage of MDA-ALB-IVM slightly increased after follow-up in Kabale but was the same before and after follow-up in Kisoro.*

*Abbreviations: MDA-ALB, mass drug administration with albendazole alone; MDA-ALB-IVM, mass drug administration with the combination of albendazole and ivermectin.*

# **Table J** Treatment time in schools (all ages)

| **District** | **Arm** | **Number of children (all ages) treated** | **Treatment time in schools** | | **Average time per 100 children (all ages) treated** |
| --- | --- | --- | --- | --- | --- |
|  |  |  | **Mean** | **Min - max** |  |
| Kabale | MDA-ALB | 2,427 | 2 h 14 min | 0 h 43 min - 3 h 37 min | 0 h 28 min 19 s |
|  | MDA-ALB-IVM | 1,374 | 4 h 30 min | 1 h 40 min - 7 h 34 min | 1 h 32 min 58 s |
| Kisoro | MDA-ALB | 3,205 | 4 h 24 min | 1 h 32 min - 8 h 43 min | 0 h 41 min 19 s |
|  | MDA-ALB-IVM | 1,761 | 4 h 54 min | 4 h 32 min - 5 h 26 min | 1 h 46 min 37 s |

*Note: Treatment time was calculated as the difference between treatment start time and stop time in schools. For MDA-ALB-IVM, the treatment time included the time required for assenting (assent and treatment were conducted sequentially); children aged eight years and above received treatment immediately after assenting, and children aged under eight years received treatment when their name and class matched their signed parental consent forms. Time per 100 children treated = treatment time / number of eligible children (all ages, including those aged <5 years and ≥ 15 years) treated * 100.*

# **Table K** Number of children treated and treatment coverage per school

| **District** | **Arm** | **School code** | **Total number of eligible children (5-14 years)** | **Before follow-up** | | **After follow-up** | | **Number of children treated outside the eligibility age range** |
| --- | --- | --- | --- | --- | --- | --- | --- | --- |
|  |  |  |  | **Number of treated** | **Treatment coverage (%)** | **Number of treated** | **Treatment coverage (%)** |  |
| Kabale | MDA-ALB-IVM | 01 | 681 | 226 | 33.2 | 226 | 33.2 | 2 |
|  |  | 02 | 558 | 203 | 36.4 | 203 | 36.4 | 7 |
|  |  | 03 | 410 | 391 | 95.4 | 391 | 95.4 | 3 |
|  |  | 04 | 681 | 254 | 37.3 | 285 | 41.9 | 1 |
|  |  | 05 | 336 | 249 | 74.1 | 249 | 74.1 | 7 |
|  | MDA-ALB | 06 | 510 | 510 | 100.0 | 510 | 100.0 | 40 |
|  |  | 07 | 216 | 157 | 72.7 | 210 | 97.2 | 25 |
|  |  | 08 | 570 | 570 | 100.0 | 570 | 100.0 | 72 |
|  |  | 09 | 663 | 653 | 98.5 | 663 | 100.0 | 7 |
|  |  | 10 | 276 | 260 | 94.2 | 276 | 100.0 | 54 |
| Kisoro | MDA-ALB-IVM | 01 | 264 | 258 | 97.7 | 258 | 97.7 | 62 |
|  |  | 02 | 587 | 553 | 94.2 | 553 | 94.2 | 82 |
|  |  | 03 | 196 | 184 | 93.9 | 184 | 93.9 | 31 |
|  |  | 04 | 240 | 224 | 93.3 | 224 | 93.3 | 61 |
|  |  | 05 | 281 | 257 | 91.5 | 257 | 91.5 | 49 |
|  | MDA-ALB | 06 | 1,156 | 1,031 | 89.2 | 1,135 | 98.2 | 75 |
|  |  | 07 | 552 | 535 | 96.9 | 551 | 99.8 | 38 |
|  |  | 08 | 353 | 353 | 100.0 | 353 | 100.0 | 154 |
|  |  | 09 | 594 | 546 | 91.9 | 582 | 98.0 | 58 |
|  |  | 10 | 215 | 187 | 87.0 | 212 | 98.6 | 47 |

*Note: Numbers in colors show that number of treated and treatment coverage were different before and after follow-up. Red is the number of treated and coverage before follow-up while blue is after follow-up. Children treated outside the eligibility age range were children aged 1-4 years and 15 years and above.*

*Abbreviations: MDA-ALB, mass drug administration with albendazole alone; MDA-ALB-IVM, mass drug administration with the combination of albendazole and ivermectin.*

# **Table L** Pilot-based cost per child treated ($) by cost categories and sub-categories

| **Cost category / sub-category** | **Kabale** | | **Kisoro** | |
| --- | --- | --- | --- | --- |
|  | **MDA-ALB** | **MDA-ALB-IVM** | **MDA-ALB** | **MDA-ALB-IVM** |
| **Medicines (financial)** | **0.00** | **0.26** | **0.00** | **0.23** |
| Ivermectin | 0.00 | 0.26 | 0.00 | 0.23 |
| **Medicines (opportunity)** | **0.04** | **0.04** | **0.04** | **0.04** |
| Albendazole | 0.04 | 0.04 | 0.04 | 0.04 |
| **Personnel (financial)** | **0.87** | **3.47** | **0.79** | **3.81** |
| Allowances | 0.29 | 1.06 | 0.34 | 1.46 |
| Per diems | 0.58 | 2.41 | 0.45 | 2.35 |
| **Transport (financial)** | **0.46** | **1.86** | **0.49** | **2.24** |
| Car hiring | 0.22 | 1.01 | 0.19 | 1.11 |
| Fuel | 0.21 | 0.72 | 0.24 | 0.89 |
| Transport refund | 0.03 | 0.13 | 0.05 | 0.24 |
| **Supplies and services (financial)** | **0.46** | **1.65** | **0.53** | **2.60** |
| Services | 0.40 | 1.30 | 0.41 | 1.54 |
| Stationery | 0.06 | 0.11 | 0.05 | 0.11 |
| Radio announcements | 0.00 | 0.00 | 0.08 | 0.72 |
| Dose poles | 0.00 | 0.06 | 0.00 | 0.05 |
| Other supplies | 0.00 | 0.18 | 0.00 | 0.17 |
| **Overheads (financial)** | **0.18** | **0.70** | **0.18** | **0.86** |
| **Total (financial and opportunity) costs** | **2.00** | **7.97** | **2.03** | **9.79** |
| Total financial costs | 1.96 | 7.93 | 2.00 | 9.75 |
| Total opportunity costs | 0.04 | 0.04 | 0.04 | 0.04 |

*Note: Cost per child treated was calculated as the pilot-based costs divided by the number of eligible children (aged 5-14 years) treated. Services included hotel packages (catering), projector renting, and airtime (Table D Cost categories and sub-categories).*

*Abbreviations: MDA-ALB, mass drug administration with albendazole alone; MDA-ALB-IVM, mass drug administration with the combination of albendazole and ivermectin.*

# **Table M** Scenario analysis (difference in % compared with the pilot-based costs)

| **District** | **Pilot-based / scenario-adjusted** | **Total costs ($)** | | **Incremental costs ($)** | **Cost per child treated ($)** | |
| --- | --- | --- | --- | --- | --- | --- |
|  |  | **MDA-ALB** | **MDA-ALB-IVM** |  | **MDA-ALB** | **MDA-ALB-IVM** |
| Kabale | Pilot-based | 4,458 (-) | 10,793 (-) | 6,335 (-) | 2.00 (-) | 7.97 (-) |
|  | Scenario-adjusted | 4,435 (-1%) | 8,828 (-18%) | 4,393 (-31%) | 1.99 (-1%) | 6.52 (-18%) |
|  | Scenario-adjusted + increasing the number of children treated in MDA-ALB-IVM to the same number (2,229) in MDA-ALB | 4,435 (-1%) | 9,076 (-16%) | 4,641 (-27%) | 1.99 (-1%) | 4.07 (-49%) |
| Kisoro | Pilot-based | 5,765 (-) | 14,445 (-) | 8,681 (-) | 2.03 (-) | 9.79 (-) |
|  | Scenario-adjusted | 5,750 (0%) | 11,277 (-22%) | 5,528 (-36%) | 2.03 (0%) | 7.64 (-22%) |
|  | Scenario-adjusted + increasing the number of children treated in MDA-ALB-IVM to the same number (2,833) in MDA-ALB | 5,750 (0%) | 11,663 (-20%) | 5,913 (-32%) | 2.03 (0%) | 4.11 (-58%) |

*Note: Difference in % was comparing the adjusted costs to the pilot-based costs. E.g., (scenario-adjusted costs – pilot-based costs) / pilot-based costs × 100%. Total and incremental costs refer to the total and incremental financial and opportunity costs. Cost per child treated was calculated as total (financial and opportunity costs) divided by the number of eligible children treated.*

*For scenario-adjusted + increasing the number of children treated in MDA-ALB-IVM to the same number (2,229) in MDA-ALB, the total costs also included the increased medicine costs for the increased number children treated.*

*Abbreviations: MDA-ALB, mass drug administration with albendazole alone; MDA-ALB-IVM, mass drug administration with the combination of albendazole and ivermectin.*

# **Table N** Scenario-adjusted total and incremental costs ($) by activities and cost categories

| **Activity / cost category** | **Kabale** | | | **Kisoro** | | |
| --- | --- | --- | --- | --- | --- | --- |
|  | **Total costs (%)** | | **Incremental costs (%)** | **Total costs (%)** | | **Incremental costs (%)** |
|  | **MDA-ALB** | **MDA-ALB-IVM** |  | **MDA-ALB** | **MDA-ALB-IVM** |  |
| **Advocacy (financial)** | **425 (9.6)** | **425 (4.8)** | **0 (0.0)** | **425 (7.4)** | **425 (3.8)** | **0 (0.0)** |
| Personnel | 200 (4.5) | 200 (2.3) | 0 (0.0) | 200 (3.5) | 200 (1.8) | 0 (0.0) |
| Transport | 226 (5.1) | 226 (2.6) | 0 (0.0) | 226 (3.9) | 226 (2.0) | 0 (0.0) |
| **Training (financial)** | **2,212 (49.9)** | **4,255 (48.2)** | **2,042 (46.5)** | **2,777 (48.3)** | **5,347 (47.4)** | **2,570 (46.5)** |
| Personnel | 941 (21.2) | 1,862 (21.1) | 921 (21.0) | 1,114 (19.4) | 2,208 (19.6) | 1,094 (19.8) |
| Transport | 311 (7.0) | 546 (6.2) | 235 (5.3) | 434 (7.5) | 768 (6.8) | 334 (6.0) |
| Supplies and services | 960 (21.6) | 1,847 (20.9) | 887 (20.2) | 1,230 (21.4) | 2,372 (21.0) | 1,142 (20.7) |
| **Sensitization (financial)** | **0 (0.0)** | **0 (0.0)** | **0 (0.0)** | **213 (3.7)** | **639 (5.7)** | **426 (7.7)** |
| Supplies and services | 0 (0.0) | 0 (0.0) | 0 (0.0) | 213 (3.7) | 639 (5.7) | 426 (7.7) |
| **Drug distribution (financial)** | **1,315 (29.7)** | **3,329 (37.7)** | **2,014 (45.8)** | **1,712 (29.8)** | **3,821 (33.9)** | **2,109 (38.1)** |
| Medicines (IVM) | 0 (0.0) | 348 (3.9) | 348 (7.9) | 0 (0.0) | 346 (3.1) | 346 (6.3) |
| Personnel | 772 (17.4) | 1,128 (12.8) | 357 (8.1) | 934 (16.2) | 1,413 (12.5) | 479 (8.7) |
| Transport | 482 (10.9) | 1,469 (16.6) | 987 (22.5) | 711 (12.4) | 1,664 (14.8) | 953 (17.2) |
| Supplies and services | 62 (1.4) | 384 (4.4) | 322 (7.3) | 67 (1.2) | 397 (3.5) | 330 (6.0) |
| **Drug distribution (opportunity)** | **86 (1.9)** | **52 (0.6)** | **-34 (-0.8)** | **110 (1.9)** | **57 (0.5)** | **-53 (-1.0)** |
| Medicines (ALB) | 86 (1.9) | 52 (0.6) | -34 (-0.8) | 110 (1.9) | 57 (0.5) | -53 (-1.0) |
| **Overheads (financial)** | **395 (8.9)** | **766 (8.7)** | **371 (8.4)** | **513 (8.9)** | **989 (8.8)** | **476 (8.6)** |
| **Total (financial and opportunity) costs** | **4,435 (100.0)** | **8,828 (100.0)** | **4,393 (100.0)** | **5,750 (100.0)** | **11,277 (100.0)** | **5,528 (100.0)** |
| Total financial costs | 4,348 (98.1) | 8,775 (99.4) | 4,427 (100.8) | 5,640 (98.1) | 11,221 (99.5) | 5,580 (101.0) |
| Total opportunity costs | 86 (1.9) | 52 (0.6) | -34 (-0.8) | 110 (1.9) | 57 (0.5) | -53 (-1.0) |

*Note: Percentages (%) in parentheses were calculated relative to the total (financial and opportunity) costs for each arm or the total incremental (financial and opportunity) costs. A negative incremental cost or percentage indicates lower cost for a given activity or category in MDA-ALB-IVM compared with MDA-ALB.*

*Abbreviations: MDA-ALB, mass drug administration with albendazole alone; MDA-ALB-IVM, mass drug administration with the combination of albendazole and ivermectin.*

# **Table O** Scenario-adjusted total and incremental costs ($) by cost categories and sub-categories

| **Cost category / sub-category** | **Kabale** | | | **Kisoro** | | |
| --- | --- | --- | --- | --- | --- | --- |
|  | **Total costs** | | **Incremental costs (%)** | **Total costs** | | **Incremental costs (%)** |
|  | **MDA-ALB** | **MDA-ALB-IVM** |  | **MDA-ALB** | **MDA-ALB-IVM** |  |
| **Medicines (financial)** | **0 (0.0)** | **348 (3.9)** | **348 (7.9)** | **0 (0.0)** | **346 (3.1)** | **346 (6.3)** |
| Ivermectin | 0 (0.0) | 348 (3.9) | 348 (7.9) | 0 (0.0) | 346 (3.1) | 346 (6.3) |
| **Medicines (opportunity)** | **86 (1.9)** | **52 (0.6)** | **-34 (-0.8)** | **110 (1.9)** | **57 (0.5)** | **-53 (-1.0)** |
| Albendazole | 86 (1.9) | 52 (0.6) | -34 (-0.8) | 110 (1.9) | 57 (0.5) | -53 (-1.0) |
| **Personnel (financial)** | **1,912 (43.1)** | **3,190 (36.1)** | **1,278 (29.1)** | **2,248 (39.1)** | **3,821 (33.9)** | **1,573 (28.5)** |
| Allowances | 648 (14.6) | 1,180 (13.4) | 532 (12.1) | 970 (16.9) | 1,705 (15.1) | 735 (13.3) |
| Per diems | 1,264 (28.5) | 2,009 (22.8) | 745 (17.0) | 1,278 (22.2) | 2,116 (18.8) | 838 (15.2) |
| **Transport (financial)** | **1,019 (23.0)** | **2,241 (25.4)** | **1,222 (27.8)** | **1,370 (23.8)** | **2,657 (23.6)** | **1,287 (23.3)** |
| Car hiring | 483 (10.9) | 1,367 (15.5) | 884 (20.1) | 539 (9.4) | 1,178 (10.4) | 639 (11.6) |
| Fuel | 461 (10.4) | 725 (8.2) | 264 (6.0) | 679 (11.8) | 1,142 (10.1) | 462 (8.4) |
| Transport refund | 75 (1.7) | 149 (1.7) | 75 (1.7) | 152 (2.6) | 338 (3.0) | 186 (3.4) |
| **Supplies and services (financial)** | **1,022 (23.0)** | **2,231 (25.3)** | **1,209 (27.5)** | **1,510 (26.3)** | **3,408 (30.2)** | **1,898 (34.3)** |
| Services | 892 (20.1) | 1,757 (19.9) | 865 (19.7) | 1,152 (20.0) | 2,268 (20.1) | 1,115 (20.2) |
| Stationery | 129 (2.9) | 150 (1.7) | 22 (0.5) | 143 (2.5) | 164 (1.5) | 22 (0.4) |
| Radio announcements | 0 (0.0) | 0 (0.0) | 0 (0.0) | 213 (3.7) | 639 (5.7) | 426 (7.7) |
| Dose poles | 0 (0.0) | 80 (0.9) | 80 (1.8) | 0 (0.0) | 80 (0.7) | 80 (1.4) |
| Other supplies | 2 (0.0) | 244 (2.8) | 242 (5.5) | 2 (0.0) | 257 (2.3) | 256 (4.6) |
| **Overheads (financial)** | **395 (8.9)** | **766 (8.7)** | **371 (8.4)** | **513 (8.9)** | **989 (8.8)** | **476 (8.6)** |
| **Total (financial and opportunity) costs** | **4,435 (100.0)** | **8,828 (100.0)** | **4,393 (100.0)** | **5,750 (100.0)** | **11,277 (100.0)** | **5,528 (100.0)** |
| Total financial costs | 4,348 (98.1) | 8,775 (99.4) | 4,427 (100.8) | 5,640 (98.1) | 11,221 (99.5) | 5,580 (101.0) |
| Total opportunity costs | 86 (1.9) | 52 (0.6) | -34 (-0.8) | 110 (1.9) | 57 (0.5) | -53 (-1.0) |

*Note: Percentages (%) in parentheses were calculated relative to the total (financial and opportunity) costs for each arm or the total incremental (financial and opportunity) costs. A negative incremental cost or percentage indicates lower cost for a given category or sub-category in MDA-ALB-IVM compared with MDA-ALB. Services included hotel packages (catering), projector renting, and airtime (Table D Cost categories and sub-categories).*

*Abbreviations: MDA-ALB, mass drug administration with albendazole alone; MDA-ALB-IVM, mass drug administration with the combination of albendazole and ivermectin.*

# **Table P** Scenario-adjusted cost per child treated ($) by activities and cost categories

| **Activity / cost category** | **Kabale** | | **Kisoro** | |
| --- | --- | --- | --- | --- |
|  | **MDA-ALB** | **MDA-ALB-IVM** | **MDA-ALB** | **MDA-ALB-IVM** |
| **Advocacy (financial)** | **0.19** | **0.31** | **0.15** | **0.29** |
| Personnel | 0.09 | 0.15 | 0.07 | 0.14 |
| Transport | 0.10 | 0.17 | 0.08 | 0.15 |
| **Training (financial)** | **0.99** | **3.14** | **0.98** | **3.62** |
| Personnel | 0.42 | 1.37 | 0.39 | 1.50 |
| Transport | 0.14 | 0.40 | 0.15 | 0.52 |
| Supplies and services | 0.43 | 1.36 | 0.43 | 1.61 |
| **Sensitization (financial)** | **0.00** | **0.00** | **0.08** | **0.43** |
| Supplies and services | 0.00 | 0.00 | 0.08 | 0.43 |
| **Drug distribution (financial)** | **0.59** | **2.46** | **0.60** | **2.59** |
| Medicines (IVM) | 0.00 | 0.26 | 0.00 | 0.21 |
| Personnel | 0.35 | 0.83 | 0.33 | 0.96 |
| Transport | 0.22 | 1.08 | 0.25 | 1.13 |
| Supplies and services | 0.03 | 0.28 | 0.02 | 0.27 |
| **Drug distribution (opportunity)** | **0.04** | **0.04** | **0.04** | **0.04** |
| Medicines (ALB) | 0.04 | 0.04 | 0.04 | 0.04 |
| **Overheads (financial)** | **0.18** | **0.57** | **0.18** | **0.67** |
| **Total (financial and opportunity) costs** | **1.99** | **6.52** | **2.03** | **7.64** |
| Total financial costs | 1.95 | 6.48 | 1.99 | 7.60 |
| Total opportunity costs | 0.04 | 0.04 | 0.04 | 0.04 |

*Note: Cost per child treated was calculated as the scenario-adjusted costs divided by the number of eligible children (aged 5-14 years) treated.*

*Abbreviations: MDA-ALB, mass drug administration with albendazole alone; MDA-ALB-IVM, mass drug administration with the combination of albendazole and ivermectin.*

# **Table Q** Scenario-adjusted cost per child treated ($) by cost categories and sub-categories

| **Cost category / sub-category** | **Kabale** | | **Kisoro** | |
| --- | --- | --- | --- | --- |
|  | **MDA-ALB** | **MDA-ALB-IVM** | **MDA-ALB** | **MDA-ALB-IVM** |
| **Medicines (financial)** | **0.00** | **0.26** | **0.00** | **0.23** |
| Ivermectin | 0.00 | 0.26 | 0.00 | 0.23 |
| **Medicines (opportunity)** | **0.04** | **0.04** | **0.04** | **0.04** |
| Albendazole | 0.04 | 0.04 | 0.04 | 0.04 |
| **Personnel (financial)** | **0.86** | **2.36** | **0.79** | **2.59** |
| Allowances | 0.29 | 0.87 | 0.34 | 1.15 |
| Per diems | 0.57 | 1.48 | 0.45 | 1.43 |
| **Transport (financial)** | **0.46** | **1.65** | **0.48** | **1.80** |
| Car hiring | 0.22 | 1.01 | 0.19 | 0.80 |
| Fuel | 0.21 | 0.54 | 0.24 | 0.77 |
| Transport refund | 0.03 | 0.11 | 0.05 | 0.23 |
| **Supplies and services (financial)** | **0.46** | **1.65** | **0.53** | **2.31** |
| Services | 0.40 | 1.30 | 0.41 | 1.54 |
| Stationery | 0.06 | 0.11 | 0.05 | 0.11 |
| Radio announcements | 0.00 | 0.00 | 0.08 | 0.43 |
| Dose poles | 0.00 | 0.06 | 0.00 | 0.05 |
| Other supplies | 0.00 | 0.18 | 0.00 | 0.17 |
| **Overheads (financial)** | **0.18** | **0.57** | **0.18** | **0.67** |
| **Total (financial and opportunity) costs** | **1.99** | **6.52** | **2.03** | **7.64** |
| Total financial costs | 1.95 | 6.48 | 1.99 | 7.60 |
| Total opportunity costs | 0.04 | 0.04 | 0.04 | 0.04 |

*Note: Cost per child treated was calculated as the scenario-adjusted costs divided by the number of eligible children (aged 5-14 years) treated. Services included hotel packages (catering), projector renting, and airtime (Table D Cost categories and sub-categories).*

*Abbreviations: MDA-ALB, mass drug administration with albendazole alone; MDA-ALB-IVM, mass drug administration with the combination of albendazole and ivermectin.*

# **Table R** Sensitivity analyses (difference in % compared with the pilot-based cost per child treated)

| **Strategy** | **Kabale** | | **Kisoro** | |
| --- | --- | --- | --- | --- |
|  | **MDA-ALB ($)** | **MDA-ALB-IVM ($)** | **MDA-ALB ($)** | **MDA-ALB-IVM ($)** |
| Pilot-based cost per child treated | 2.00 (-) | 7.97 (-) | 2.03 (-) | 9.79 (-) |
| ALB at half price | 1.98 (-1%) | 7.95 (0%) | 2.02 (0%) | 9.77 (0%) |
| ALB at double price | 2.04 (2%) | 8.01 (1%) | 2.07 (2%) | 9.83 (0%) |
| IVM at half price | 2.00 (-) | 7.84 (-2%) | 2.03 (-) | 9.67 (-1%) |
| IVM at double price | 2.00 (-) | 8.23 (3%) | 2.03 (-) | 10.02 (2%) |
| 10% decrease in coverage | 2.22 (11%) | 8.20 (3%) | 2.26 (11%) | 10.93 (12%) |
| 20% decrease in coverage | 2.50 (25%) | 8.46 (6%) | 2.55 (26%) | 12.41 (27%) |
| 10% increase in coverage (Kabale, MDA-ALB-IVM arm) | 2.00 (-) | 6.96 (-13%) | 2.03 (-) | 9.79 (0%) |
| 20% increase in coverage (Kabale, MDA-ALB-IVM arm) | 2.00 (-) | 6.19 (-22%) | 2.03 (-) | 9.79 (0%) |

*Note: Cost per child treated was calculated as the total (financial and opportunity) costs divided by the number of eligible children (aged 5-14 years) treated.*

*Abbreviations: ALB, albendazole; ALB-IVM, combination of albendazole and ivermectin; MDA-ALB, mass drug administration with albendazole alone; MDA-ALB-IVM, mass drug administration with the combination of albendazole and ivermectin.*

# **Figure A** Tornado plots of scenario-adjusted sensitivity analyses


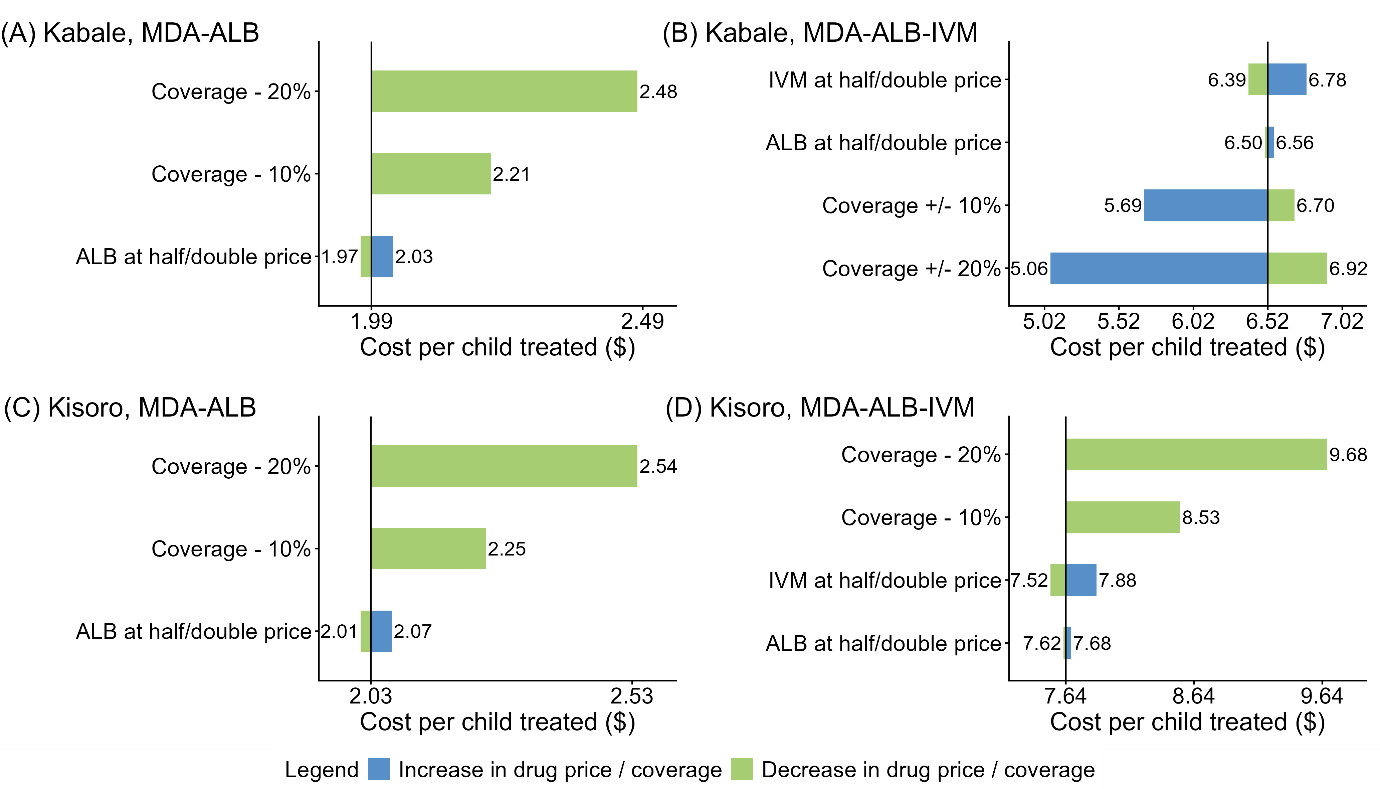


**Figure A** Tornado plots of scenario-adjusted sensitivity analyses

*Note: Bars show the estimated scenario-adjusted cost per child treated (aged 5-14 years) under various sensitivity scenarios in different intervention settings. Cost per child treated was calculated as the total (financial and opportunity) scenario-adjusted costs divided by the number of eligible children (aged 5-14 years) treated. Bars in blue represent the estimated cost per child treated after increasing drug price or treatment coverage, while bars in green represent the estimated cost per child treated after decreasing drug price or treatment coverage. The central value in each panel reflects the base-case estimate* *without varying drug price or treatment coverage, while bars indicate how this estimate shifts under alternative assumptions for drug pricing and treatment coverage. Wider bars represent greater sensitivity to the parameter in question, highlighting which assumptions most influence the estimates of cost per child treated.*

*Abbreviations: ALB, albendazole; IVM, ivermectin; MDA-ALB, mass drug administration with albendazole alone; MDA-ALB-IVM, mass drug administration with the combination of albendazole and ivermectin.*

1. District neglect tropic disease (NTD) focal persons are personnel at district who coordinate, facilitate and support NTD-related work, such as mass drug administration. [↑](#footnote-ref-1)
2. In Uganda, village health team (VHT) is based at village level; there can be one to few VHT members per village. VHT coordinators are at sub-county level (one VHT coordinator per sub-county. Both VHT members and coordinators are called VHTs. In this study, One VHT means either one VHT member or coordinator. [↑](#footnote-ref-2)
